# Clinical and radiological characteristics of Mpox pneumonia in immunosuppressed HIV-infected patients: a multicenter study from China

**DOI:** 10.3389/fmicb.2025.1705001

**Published:** 2025-11-20

**Authors:** Wei Wang, Zhongkai Zhou, Chunwang Yuan, Fuxiang Wang, Yue Yin, Lingling Zhao, Yanbin Shi, Budong Chen, Hongjun Li

**Affiliations:** 1 Department of Radiology, Beijing Youan Hospital, Capital Medical University, Beijing, China; 2 Department of Interventional Radiology, Beijing Youan Hospital, Capital Medical University, Beijing, China; 3 Department of Infectious Diseases and Critical Care Medicine, Shenzhen Third People’s Hospital, Shenzhen, China; 4 Department of Radiology, The Sixth Peoples Hospital of Zhengzhou, Zhengzhou, China

**Keywords:** monkeypox virus, human immunodeficiency virus, Mpox pneumonia nodules, thin-section computed tomography, immunosuppression

## Abstract

**Background:**

In people living with HIV, particularly with low CD4^+^ T-cell counts, monkeypox virus (Mpox) can disseminate to visceral organs, leading to severe outcomes. Mpox pneumonia is a prominent severe phenotype, but systematic reports from China are scarce.

**Methods:**

This multicenter retrospective study analyzed 41 HIV-infected patients with Mpox (June 2022–February 2025), divided into a pneumonia group (*n* = 21) and a control group (*n* = 20). Clinical, laboratory, and thin-section chest computed tomography (CT) findings, multisystem involvement, outcomes, and the roles of immune status and antiretroviral therapy (ART) adherence were compared.

**Results:**

All patients were men who have sex with men. Mpox pneumonia occurred almost exclusively in patients with CD4^+^ T-cell counts <200/μL. Thin-section CT revealed multiple, randomly distributed, well-demarcated, non-enhancing nodules (2–36 mm), consistent with hematogenous necrotizing lesions. All pneumonia cases had ≥2 organ systems involved (e.g., proctitis, necrotic skin lesions, intestinal obstruction, sepsis). Compared with controls, pneumonia patients showed more frequent inflammation and organ injury markers (elevated C-reactive protein, procalcitonin, D-dimer, creatinine, and anemia). The case fatality rate was 38.1% (8/21) in the pneumonia group versus 0% in controls. Patients with consistent ART recovered, whereas all deaths occurred in those untreated or with irregular ART adherence.

**Conclusion:**

Mpox pneumonia in HIV-infected individuals primarily affects those with advanced immunosuppression, presenting with disseminated necrotizing pulmonary nodules, multisystem involvement, and high mortality. Consistent ART markedly improves prognosis, highlighting the need for early identification of high-risk patients and integrated management—including sustained ART and targeted Mpox treatment—to reduce severe outcomes.

## Introduction

Monkeypox virus (Mpox), a member of the Orthopoxvirus genus, was historically endemic to tropical rainforest regions of Central and West Africa, where it primarily spread through zoonotic transmission, with limited human-to-human transmission reported (Kozlov, 2022). Since May 2022, Mpox has rapidly spread worldwide and was declared a Public Health Emergency of International Concern (PHEIC) by the World Health Organization (WHO) later that year (World Health Organization, 2023). As of July 2024, more than 100,000 confirmed cases and 223 deaths had been reported across 121 countries (Jadhav et al., 2025).

The current outbreak demonstrates a transmission pattern distinct from previous epidemics, with predominant spread through close skin-to-skin or mucosal contact, particularly among men who have sex with men (MSM), where a high proportion of cases involve individuals living with HIV (Vaughan et al., 2022). A multinational cohort study published in The Lancet reported that approximately 47% of Mpox patients were co-infected with HIV, with over one-quarter having CD4^+^ T cell counts below 200 cells/μL, suggesting that immunological status plays a critical role in disease progression (Mitjà et al., 2023). Unlike immunocompetent individuals who typically present with mild and self-limiting cutaneous lesions, Mpox infection in profoundly immunosuppressed patients is more likely to manifest as a disseminated, multi-organ syndrome with severe clinical features such as necrotic skin lesions, proctitis, meningoencephalitis, and necrotizing pneumonia, all associated with substantially higher mortality rates (Mitjà et al., 2023; Deb et al., 2025; Yakubovsky et al., 2023; Garcia et al., 2024).

While cutaneous, gastrointestinal, and neurological involvement has been well-documented, pulmonary complications—despite being a major target of systemic Mpox dissemination—remain poorly characterized in terms of imaging features, pathogenesis, and clinical outcomes. Autopsy data have shown that in patients with AIDS, Mpox can disseminate hematogenously to the lungs, leading to nodular necrosis, thrombotic vasculopathy, and alveolar parenchymal damage, with the highest mortality observed in individuals with CD4^+^ T cell counts <200 cells/μL (Mitjà et al., 2023; Ritter et al., 2024). Therefore, systematic investigation of pulmonary Mpox is critical for the early identification of high-risk individuals and the development of personalized intervention strategies.

Given the unique advantages of thin-section chest computed tomography (CT) (≤1.0 mm) and dynamic contrast-enhanced CT (DCE-CT) in evaluating pulmonary lesion morphology, distribution, and vascular supply, this multicenter study aimed to systematically characterize the typical imaging features of Mpox pneumonia in the context of immunosuppression, to explore its dissemination mechanisms and associations with systemic injury, and to differentiate it radiologically from common HIV-related opportunistic pneumonias. In addition, we analyzed the relationship between imaging characteristics and CD4^+^ T-cell counts, and evaluated the association of antiretroviral therapy (ART) adherence with clinical outcomes, with the goal of providing evidence-based guidance for early identification, risk stratification, and intervention in high-risk populations.

## Materials and methods

### Participants

This study was conducted in strict accordance with the ethical principles outlined in the Declaration of Helsinki and was approved by the Ethics Committees of Beijing You’an Hospital, Capital Medical University, and Zhengzhou Sixth People’s Hospital (Approval Nos. LL-2023-035-K and IEC-KY-2023-19). Written informed consent was obtained from all participants prior to enrollment. For data derived exclusively from anonymized medical records, the requirement for informed consent was waived by the ethics committees. The study period spanned from June 2022 to February 2025. Eligible participants were HIV-positive individuals who visited or were referred to the participating centers during this period and were diagnosed with Mpox infection via real-time reverse transcription polymerase chain reaction (RT–PCR). Mpox diagnosis was confirmed by the detection of Mpox viral nucleic acid in samples from at least three distinct anatomical sites (e.g., skin lesions, pharynx, anus, or blood), thereby enhancing pathogen-specific diagnostic accuracy. Inclusion criteria for pneumonia patients were as follows: All participants had a confirmed HIV diagnosis. Due to their high risk of pulmonary opportunistic infections, individuals presenting with respiratory symptoms (e.g., cough, sputum production, dyspnea, chest pain) or elevated inflammatory markers on admission underwent thin-section chest CT evaluation.

The diagnosis of Mpox pneumonia was established using a two-step screening process: Step 1-Initial screening for pneumonia: Based on the 2019 ATS/IDSA guidelines for the diagnosis of community-acquired pneumonia (CAP), patients were preliminarily identified as having pneumonia if they met all of the following three criteria: (Kozlov, 2022) at least one respiratory or systemic symptom (e.g., cough, fever, dyspnea, or pleuritic chest pain); (World Health Organization, 2023) abnormal findings on physical examination (e.g., rales) or elevated laboratory markers (e.g., CRP or procalcitonin); and (Jadhav et al., 2025) new-onset consolidation, ground-glass opacity, or infiltration observed on chest CT (Metlay et al., 2019). Step 2-Confirmation of Mpox pneumonia: On top of initial screening, Mpox pneumonia was diagnosed if all of the following were present: (Kozlov, 2022) Mpox nucleic acid positivity from at least three anatomical sites; (World Health Organization, 2023) thin-section chest CT demonstrating hematogenous disseminated solid nodules with well-defined margins and random distribution; and (Jadhav et al., 2025) an etiologic assessment based on pathogen detection in serum, sputum, or bronchoalveolar lavage (BAL) to rule out other definite opportunistic infections as causes of these hematogenously disseminated nodules.

Although typical Mpox-related pulmonary nodules exhibit relatively specific imaging characteristics, the possibility of mixed-pathogen co-infection should be considered in immunosuppressed individuals. For cases with atypical or overlapping imaging features, DCE-CT may be performed to evaluate vascularity when clinically indicated; in selected cases, CT-guided fine-needle aspiration biopsy (FNAB) may be performed to assist in differential diagnosis.

In immunocompromised individuals, additional pulmonary infections (such as bacterial or fungal pneumonia) may coexist alongside the typical Mpox-related nodules, with imaging manifestations including patchy consolidation, ground-glass opacities, or a “tree-in-bud” pattern.

**Figure 1:**
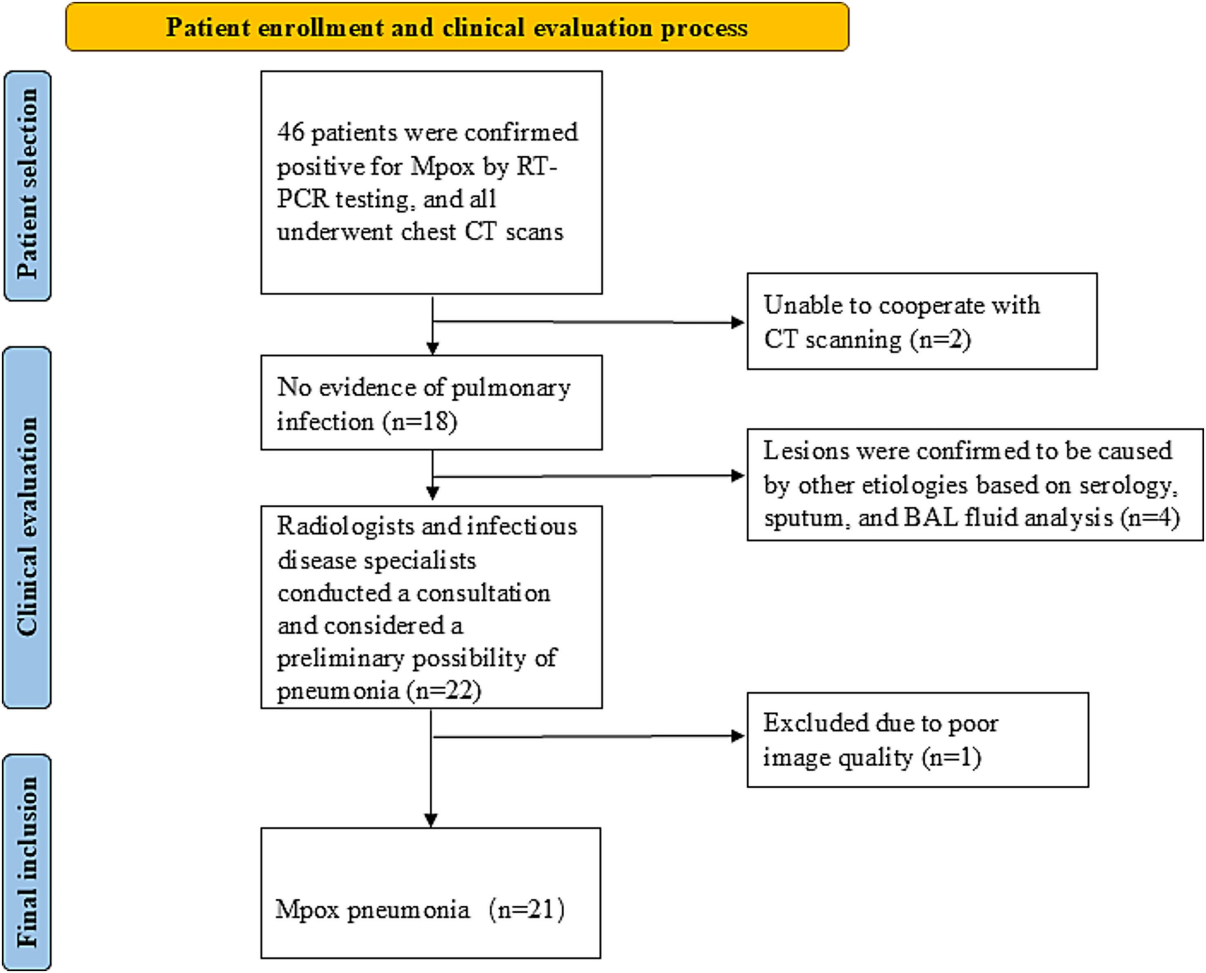
Flowchart shows details of patient enrollment in study. RT-PCR, real-time fluorescence reverse transcription polymerase chain reaction; BAL, bronchoalveolar lavage.

Ultimately, 21 patients who met the above criteria were included in the Mpox pneumonia group (see flowchart in Figure 1). In addition, 20 HIV-infected individuals with confirmed Mpox infection but no pulmonary involvement were randomly selected as controls. All participants underwent high-quality thin-section chest CT scans to ensure consistency and comparability of imaging data for subsequent analysis.

### Demographic, clinical, and immunological data collection

For all patients, if the exact timing of high-risk sexual behavior could not be recalled, the onset of initial symptoms was used as the clinical starting point. Demographic information, epidemiological data, HIV diagnosis and treatment status, clinical manifestations, comorbidities, opportunistic infections, and final outcomes were collected using a standardized electronic medical record system. HIV status and ART adherence prior to admission were categorized into three groups: (Kozlov, 2022) previously diagnosed and consistently receiving ART; (World Health Organization, 2023) newly diagnosed with HIV and not yet initiated or only recently initiated ART; and (Jadhav et al., 2025) previously diagnosed but with poor or no ART adherence. Information on ART adherence was primarily extracted from inpatient electronic medical records and patient-reported history at admission, and was further verified using HIV follow-up archives in selected centers. Comorbidities included syphilis, previous pulmonary tuberculosis, hypertension, hepatitis B virus infection, and psoriasis. Opportunistic infections were diagnosed based on microbiological evidence (e.g., culture or PCR testing of sputum, blood, or other sterile sites) and/or radiological findings. Immunological assessment included CD4^+^ T-cell counts. It is noteworthy that HIV status and ART adherence were assessed prior to admission. Following admission, all patients underwent initiation or optimization of ART and received Mpox-specific antiviral therapy, supplemented by supportive care and treatment of secondary infections as recommended by national and international guidelines (Rao et al., 2023).

A total of 41 HIV-infected individuals were included in this study. Among them, 21 patients met the diagnostic criteria for Mpox pneumonia and were assigned to the Mpox pneumonia group, while the remaining 20 patients, who had HIV/Mpox co-infection without pulmonary involvement, served as controls. Clinical data collected included systemic symptoms (such as fever, myalgia, fatigue, and lymphadenopathy), the chronological relationship between rash and systemic symptoms (rash preceding, following, or occurring simultaneously with fever), and the initial site of rash onset (genital, perianal, oral/perioral, or other regions). Systemic dissemination was defined as the involvement of at least two extrapulmonary organ systems, including: (Kozlov, 2022) necrotic skin lesions (ulcers, gangrene, or necrotizing fasciitis); (World Health Organization, 2023) respiratory system (pneumonia, pleural effusion, respiratory failure); (Jadhav et al., 2025) gastrointestinal system (oral/esophageal ulcers, proctitis, bowel obstruction, peritonitis); (Vaughan et al., 2022) nervous system (central nervous system infection, peripheral neuropathic pain); (Mitjà et al., 2023) ocular system (conjunctivitis); (Deb et al., 2025) urinary system (renal insufficiency, acute tubular necrosis, urinary tract infection); (Yakubovsky et al., 2023) systemic inflammatory response (sepsis, septic shock); and (Garcia et al., 2024) metabolic–endocrine system (electrolyte disturbances, hypoproteinemia).

Laboratory evaluations included CD4^+^ T cell count, CD4/CD8 ratio, HIV viral load, complete blood count, renal function tests, inflammatory markers, and coagulation parameters.

### Chest CT scanning parameters and procedures

Chest CT scans were performed at the two study centers using BriIIiance iCT 256 (Philips Healthcare) and Optima CT680 (GE Healthcare) spiral CT scanners, respectively. All patients were scanned in the supine position following deep inspiration with breath-hold, covering the area from lung apex to costophrenic angle. The scanning parameters were standardized as follows: tube voltage 120 kV, automatic tube current modulation, matrix size 512 × 512, slice thickness and pitch both 5 mm, with reconstructed slice thickness of 1 mm or 0.625 mm. For patients undergoing contrast-enhanced CT, 80–100 mL of nonionic iodinated contrast agent was administered intravenously at a rate of 3–4 mL/s. Arterial-phase (30–35 s) and delayed-phase (60–65 s) images were subsequently acquired.

### Imaging assessment

Two radiologists from each of the two participating centers, each with over 5 years of experience in thoracic imaging, independently evaluated all thin-section chest CT scans in a double-blind manner. In cases of disagreement, the final interpretation was determined by a senior thoracic radiologist with more than 20 years of diagnostic experience. The imaging analysis included the following aspects: lesion type, including ground-glass nodules (GGNs, defined as hazy areas of increased attenuation with preserved bronchial and vascular markings), solid nodules (homogeneous high-density opacities that obscure pulmonary vasculature and bronchi), and mixed-type nodules (containing both ground-glass and solid components). Lesion distribution was assessed across four dimensions: (Kozlov, 2022) lobar involvement (categorized as single-lobe, 2–3 lobes, or ≥4 lobes); (World Health Organization, 2023) axial distribution (classified as central or peripheral based on location relative to the lobar axis); (Jadhav et al., 2025) craniocaudal distribution (lesions above the carina were classified as upper-lung dominant and those below as lower-lung dominant); and (Vaughan et al., 2022) random distribution (defined as no obvious clustering or regional preference in any of the above dimensions). Maximum nodule diameter was categorized as <10 mm, 10–30 mm, or >30 mm. Characteristic imaging signs such as spiculation, lobulation, and halo signs were recorded. In addition to Mpox-characteristic solid nodules, other intrapulmonary inflammatory patterns were recorded to identify potential co-infections. These included consolidation, ground-glass opacities, or interstitial changes, which may indicate fungal, bacterial, tuberculous, or other viral infections. Extrapulmonary findings included mediastinal or hilar lymphadenopathy, pleural effusion, and pleural thickening. In contrast-enhanced CT analysis, regions of interest (ROIs) were placed at the same anatomical location on pre- and post-contrast images (mediastinal window). The enhancement value (ΔHU = HU post-contrast—HU pre-contrast) was calculated, and a ΔHU ≥ 15 was considered indicative of nodule enhancement (Swensen et al., 2000).

### Statistical analysis

Statistical analyses were performed using SPSS software (version 28.0, IBM). Categorical variables were expressed as frequencies (percentages). The Shapiro–Wilk test was applied to assess the normality of continuous variables. Normally distributed variables were presented as mean ± standard deviation (*x̄* ± s), while non-normally distributed variables were presented as median (interquartile range) [*M* (*IQR*)]. Group comparisons of categorical variables were performed using the *χ*^2^ test or Fisher’s exact test (when expected frequencies were <5). For ordinal categorical variables (e.g., stratified HIV status), the *χ^2^* test for trend was applied. Continuous variables were compared using the independent-samples *t*-test for normally distributed data or the Mann–Whitney *U*-test for non-normally distributed data. All *p* values were two-tailed, with *p* < 0.05 considered statistically significant. Inter-observer reliability for categorical variables was assessed using Cohen’s kappa (*κ*) and weighted Cohen’s kappa, interpreted as follows: <0.20, poor; 0.21–0.40, fair; 0.41–0.60, moderate; 0.61–0.80, substantial; and 0.81–1.00, almost perfect agreement.

## Results

### Clinical findings

A total of 41 male patients with HIV infection were enrolled, all of whom were men who have sex with men (MSM). Among them, 21 were classified into the Mpox pneumonia group and 20 into the control group. The median age in both groups was 29 years (IQR: 17–38 years in the Mpox pneumonia group; 26–38.5 years in the control group). In the Mpox pneumonia group, 8 patients (38.1%) had a prior HIV diagnosis with consistent ART, 8 (38.1%) were newly diagnosed with HIV, and 6 (28.6%) had a prior HIV diagnosis but irregular or no ART use. All patients in this group had CD4^+^ T-cell counts <200/μL. In the control group, 15 patients (75.0%) had a prior HIV diagnosis with consistent ART, and 5 (25.0%) were newly diagnosed. Nineteen patients had CD4^+^ T-cell counts ≥200/μL, while 1 patient had a count <200/μL.

Among the 22 patients with CD4^+^ T-cell counts <200/μL, seven met the criteria for incomplete immune reconstitution (IIR), defined as regular ART for ≥12 months with virological suppression but persistently low CD4^+^ counts. Six were classified as non-IIR, referring to previously diagnosed individuals with irregular ART use or prolonged ART interruption who presented with profound immunosuppression at admission. The remaining nine were late presenters, including those newly diagnosed with HIV during the current Mpox evaluation or previously diagnosed but never initiated on ART or enrolled in long-term care.

The sequence of rash and systemic symptom onset was as follows: in the Mpox pneumonia group, 2 patients (9.5%) developed systemic symptoms first, 9 (42.9%) had rash onset first, 2 (9.5%) had both simultaneously, and 8 (38.1%) presented with rash but no systemic symptoms. In the control group, 12 patients (60.0%) experienced systemic symptoms first, 3 (15.0%) developed rash first, 1 (5.0%) had simultaneous onset, and 4 (20.0%) had no systemic symptoms. Initial rash distribution: genital (66.7%) and perianal (19.0%) regions predominated in the Mpox pneumonia group; in the control group, 65.0% had genital rash and 5.0% perianal. Incidence of multisystem involvement (≥2 organ systems): 100% (21/21) in the Mpox pneumonia group versus 10.0% (2/20) in the control group. Frequently affected systems in the Mpox pneumonia group included the respiratory system (100%), gastrointestinal (66.7%), metabolic–endocrine (52.4%), urinary (33.3%), and systemic inflammatory responses (19.0%).

### Disease and treatment course

In this study, all 21 patients with Mpox pneumonia exhibited systemic dissemination (involvement of two or more extrapulmonary systems). For these critically ill patients, we employed a comprehensive, systematic intensive therapy, which included the following key components: (Kozlov, 2022) HIV infection management: ART was initiated or optimized immediately after stabilization of vital signs, based on Chinese guidelines and resistance profiles. The core goal was to suppress HIV replication and promote immune reconstitution, providing the foundation for clearing the Mpox virus and controlling concomitant infections (World Health Organization, 2023). Supportive treatment for Mpox and related complications: Mpox-specific antiviral therapy was administered, along with enteral or parenteral nutrition and professional care for skin lesions to prevent secondary infections (Jadhav et al., 2025). Targeted treatment for opportunistic infections and comorbidities: Intensive broad-spectrum or pathogen-targeted therapy was applied according to imaging and microbiological findings.

The disease duration was longer in the Mpox pneumonia group than in the control group (38.0 days vs. 16.5 days), indicating that the severe immune suppression and multi-system involvement contributed to a prolonged disease course. The treatment course was associated with early ART initiation, which was essential for patient survival.

### Outcomes

In the Mpox pneumonia group, 8 patients (38.1%) died, while 13 (61.9%) recovered and were discharged. All fatal cases occurred in individuals with profound immunosuppression, including four non-IIR patients with a prior HIV diagnosis but irregular or prolonged interruption of ART, and four late presenters newly diagnosed with HIV, indicating that delayed diagnosis and ART interruption were strongly associated with adverse clinical outcomes. In contrast, all patients on regular ART, despite incomplete immune reconstitution and multi-organ involvement, survived through intensive therapy. All patients in the control group (100%) recovered without mortality; of these, 15 (75.0%) had a prior HIV diagnosis with consistent ART, and 5 (25.0%) were newly diagnosed (see Table 1).

**Table 1:** Patient characteristic information.

Characteristic	Mpox pneumonia+	Mpox pneumonia−	*P*-value
Sex			
Male	21 (100.0)	20 (100.0)	
Age	29 (17, 38)	29 (26, 38.5)	0.169
MSM	21 (100.0)	20 (100.0)	
HIV status			0.008
Previously known PLWH currently adherent to ART	7 (33.3)	15 (75.0)	
Newly diagnosed with HIV infection	8 (38.1)	5 (25.0)	
Previously known PLWH not on ART or non-adherent	6 (28.6)	0	
Opportunistic infections (present/absent)			0.009
Present, *n* (%)	15 (71.4)	2 (10.0)	
Comorbidities (present/absent)			<0.001
Comorbidities (≥1)	18 (85.7)	10 (50.0)	
The sequence of rash and fever onset			0.006
Fever before rash	2(9.5)	12(60.0)	
Rash before fever	9(42.9)	3 (15.0)	
Rash and fever occurred simultaneously	2(9.5)	1 (5.0)	
Rash without fever	8(38.1)	4 (20.0)	
Initial rash location			0.145
Genital	14 (66.7)	13 (65.0)	
Anorectal	4 (19.0)	1 (5.0)	
Oral/perioral			
Other	3 (14.3)	6 (30.0)	
Multisystem involvement^‡^			<0.001
Necrotic skin damage	3 (14.3)	2 (10.0)	
Respiratory system	21 (100)	2 (10.0)	
Cardiovascular system	3 (14.3)	0	
Digestive system	14 (66.7)	0	
Nervous system	3 (14.3)	0	
Urinary system	7 (33.3)	2 (10.0)	
Systemic inflammatory system (sepsis/shock)	4 (19.0)	0	
Metabolic–endocrine system	11 (52.4)		
Involvement of ≥2 systems	21 (100)	2 (10.0)	
CD4 cell count <200/≥200			<0.001
<200 cells/μL	21 (100)	1 (5.0)	
Duration of illness, days, median (IQR)	38.0 (31, 100)	16.5 (10.5, 21)	<0.001
Disease outcome, Death (Yes/No)			<0.001
Recovered, *n* (%)	13 (61.9)	20 (100)	
Previously known HIV+, regular ART adherence	8 (38.1)	15 (75.0)	
Newly diagnosed HIV	3 (14.3)	5 (25.0)	
Previously known HIV+, irregular/no ART	2 (9.5)	0	
Died, *n* (%)	8(38.1)	0	
Previously known HIV+, regular ART adherence	0	0	
Newly diagnosed HIV	4 (19.0)	0	
Previously known HIV+, irregular/no ART	4 (19.0)	0	

Unless otherwise specified, data are the number of patients, with percentages in parentheses; Comorbidities included syphilis, prior tuberculosis, hypertension, chronic hepatitis B virus (HBV) infection, and psoriasis, as documented before admission. Opportunistic infections were confirmed by microbiological (e.g., sputum culture, blood culture, PCR assays) and/or radiological evidence during hospitalization.

^‡^Multisystem involvement was defined as the involvement of two or more extrapulmonary organ systems, including: Necrotic skin lesions (e.g., cutaneous ulcers, gangrene, necrotizing fasciitis); Respiratory system (e.g., pneumonia, pleural effusion, respiratory failure); Digestive system (e.g., oral or esophageal ulcers, proctitis, bowel obstruction, gut microbiota dysbiosis, peritonitis); Nervous system (e.g., central nervous system infection, peripheral neuralgia); Ocular involvement (e.g., conjunctivitis); Urinary system (e.g., renal insufficiency, acute kidney injury, urinary tract infection); Systemic inflammatory system (e.g., sepsis, septic shock); Metabolic–endocrine system (e.g., electrolyte imbalance, hypoproteinemia).

All ART adherence categories in the table refer to status before admission; all patients received ART initiation or optimization during hospitalization.

### Laboratory findings

Laboratory abnormalities in the Mpox pneumonia group were as follows: (Kozlov, 2022) Hematological parameters: leukocytosis in 12 patients (57.1%), thrombocytosis in 8 (38.1%), decreased hemoglobin in 11 (52.4%), and lymphopenia in 8 (38.1%) (World Health Organization, 2023). Renal function: elevated serum creatinine in 6 patients (28.6%) (Jadhav et al., 2025). Inflammatory markers: elevated C-reactive protein (CRP) in 14 patients (66.7%) and elevated procalcitonin (PCT) in 4 (19.0%) (Vaughan et al., 2022). Coagulation indicators: prolonged prothrombin time in 13 patients (61.9%) and elevated D-dimer in 12 (57.1%). In the control group, most laboratory parameters remained within the normal range; only 2 cases (11.1%) each showed leukocytosis and thrombocytosis, 9 (50.0%) had elevated CRP, and 1 (5.6%) showed elevated D-dimer. Overall, patients in the Mpox pneumonia group more frequently exhibited hyperinflammatory responses, coagulation abnormalities, anemia, and renal impairment, indicating a multisystem pathological involvement and highlighting a marked clinical contrast with the control group (see Table 2).

**Table 2:** Laboratory findings of Mpox pneumonia.

Laboratory test	No. normal		No. reduced		No. elevated	
	Mpox pneumonia+	Mpox pneumonia−	Mpox pneumonia+	Mpox pneumonia−	Mpox pneumonia+	Mpox pneumonia−
WBC	9 (42.9)	18 (90.0)	0	0	12 (57.1)	2 (10.0)
Platelet	13 (61.9)	18 (90.0)	0	0	8 (38.1)	2 (10.0)
Haemoglobin	10 (47.6)	19 (95.0)	11 (52.4)	1 (5.0)	0	0
Lymphocyte	13 (61.9)	17 (85.0)	8 (38.1)	0	0	3 (15.0)
Blood creatinine	15 (71.4)	20 (100.0)	6 (28.6)	0	0	0
Procalcitonin	16 (76.2)	20 (100.0)	1 (4.8)	0	4 (19.0)	0
Prothrombin	5 (23.8)	20 (100.0)	3 (14.3)	0	13 (61.9)	0
D-Dimer	5 (23.8)	19 (95.0)	4 (19.0)	0	12 (57.1)	1 (5.0)
CRP	6 (28.6)	11 (55.0)	1 (4.8)	0	14 (66.7)	9 (45.0)

Values in parentheses are percentages. WBC, white blood cell; CRP, C-reactive protein.

### CT findings

**Figure 2:**
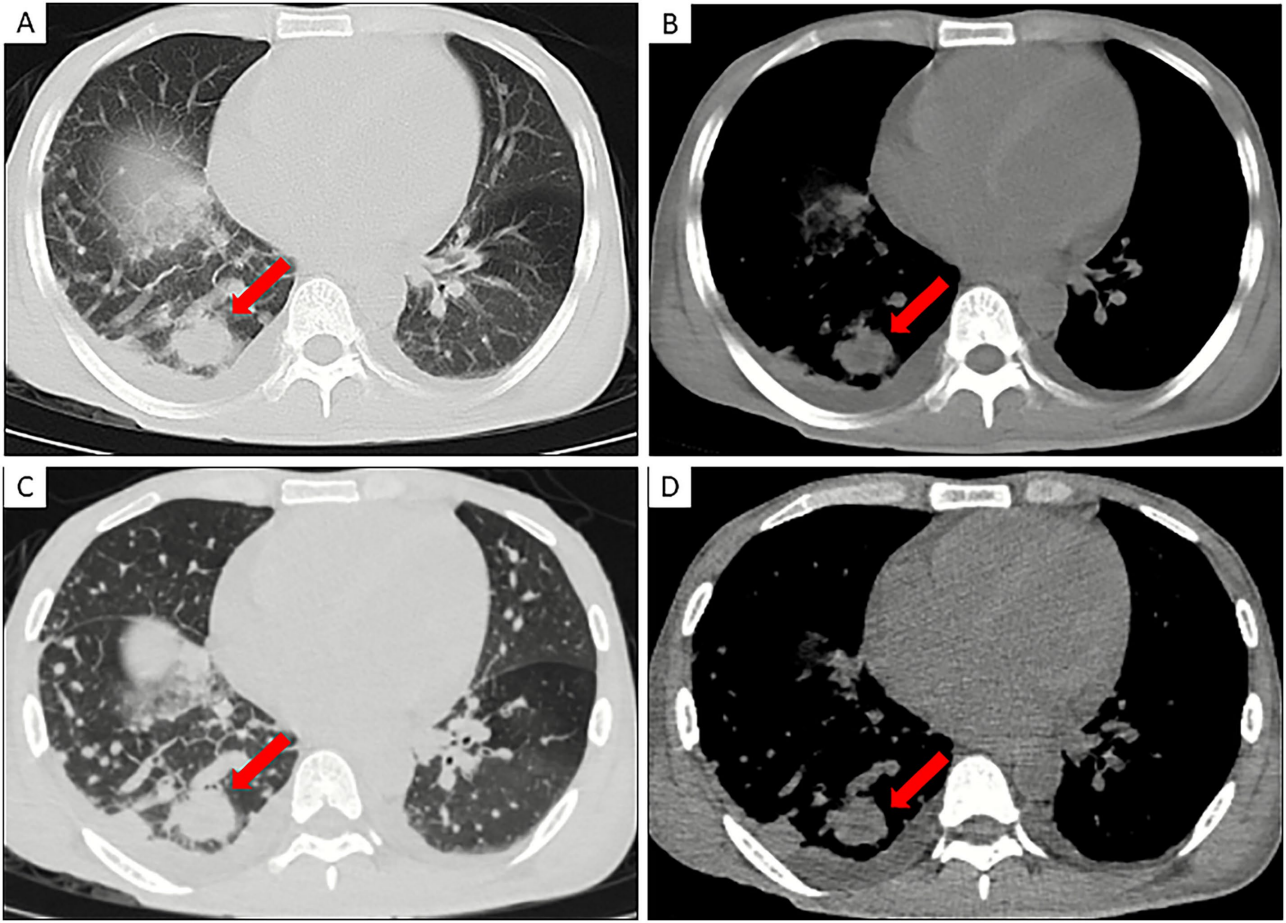
A 29-year-old male, suffering from Mpox pneumonia, initially presented with fever and a history of risky homosexual behavior. **(A,B)** CT scans in lung window **(A)** and mediastinal window **(B)** reveal a solid nodular lesion in the posterior basal segment of the right lower lobe (red arrow), measuring approximately 36 mm in diameter (considering its origin, it is still classified as a nodule rather than a mass). **(C,D)** thin-section reconstructions in lung window **(C)** and mediastinal window **(D)** show the lesion with irregular borders, characterized by conspicuous spiculation and lobulation.

In all 21 patients with HIV/Mpox pneumonia, thin-section chest CT revealed characteristic hematogenous disseminated solid nodules (Figure 2). Among the 16 patients with a documented history of high-risk homosexual contact, the median interval from exposure to the detection of pulmonary nodules was 11 days (IQR: 8–27 days). For the remaining five patients who could not recall the exact time of exposure, pulmonary nodules were identified by CT a median of 23 days (IQR: 3–29 days) after the onset of rash or systemic symptoms.

The distribution of nodules among lung lobes was as follows: left upper lobe in 10 patients (47.6%), left lower lobe in 12 (57.1%), right upper lobe in 14 (66.7%), right middle lobe in 11 (52.4%), and right lower lobe in 15 (71.4%). Based on the number of affected lobes, 4 patients (19.0%) had involvement of a single lobe, 4 (19.0%) had 2–3 lobes involved, and 13 (61.9%) showed widespread involvement of 4–5 lobes. Regarding distribution patterns, most lesions were randomly distributed without clear zonal predominance in both the vertical (90.5%, 19/21) and axial (81.0%, 17/21) planes.

Nodule diameters ranged from 2 to 36 mm, with most measuring between 10 and 30 mm. In 11 patients (52.4%), mixed ground-glass nodules coexisted with solid nodules. In 8 patients (38.1%), some nodules exhibited mild lobulation, and in 6 patients (28.6%), spiculated margins were observed. A total of 14 patients (66.7%) showed additional imaging findings suggestive of co-infection on thin-section chest CT, beyond Mpox-specific pneumonia. Specifically, 2 patients (9.5%) had small airway infection with typical “tree-in-bud” signs; 11 (52.4%) demonstrated patchy consolidation, among whom 7 (33.3%) also exhibited patchy or band-like ground-glass opacities; and 1 patient (4.8%) had concomitant tuberculosis. Extrapulmonary findings included mediastinal or hilar lymphadenopathy in 11 patients (52.4%), pleural effusion in 6 (28.6%), and pleural thickening in 2 (9.5%) (Table 3).

**Table 3:** CT Features of Mpox pneumonia.

CT finding	No.
Nodule location	
Upper left lung	10 (47.6)
Lower left lung	12 (57.1)
Upper lobe of right lung	14 (66.7)
Middle lobe of right lung	11 (52.4)
Lower lobe of right lung	15 (71.4)
No. of lobes	
1	4 (19.0)
2 or 3	4 (19.0)
4 or 5	13 (61.9)
Axial distribution	
Inner	1 (4.8)
Outer	0
Random	19 (90.5)
Longitudinal distribution	
Upper	2 (9.5)
Lower	2 (9.5)
Random	17 (81.0)
Size of the single largest lesion (mm)	
<10	0
10–30	20 (95.2)
>30	1 (4.8)
Properties of lesion	
GGO	0
Solid	21 (100)
Subsolid	11 (52.4)
Special signs	
Halo sign	0
Spiculation	6 (28.6)
Extrapulmonary manifestations	
Mediastinal and hilar lymph node enlargement	11 (52.4)
Pleural effusion	6 (28.6)
Pleural thickening	2 (9.5)

Values in parentheses are percentages; GGO, ground-glass opacity.

**Figure 3:**
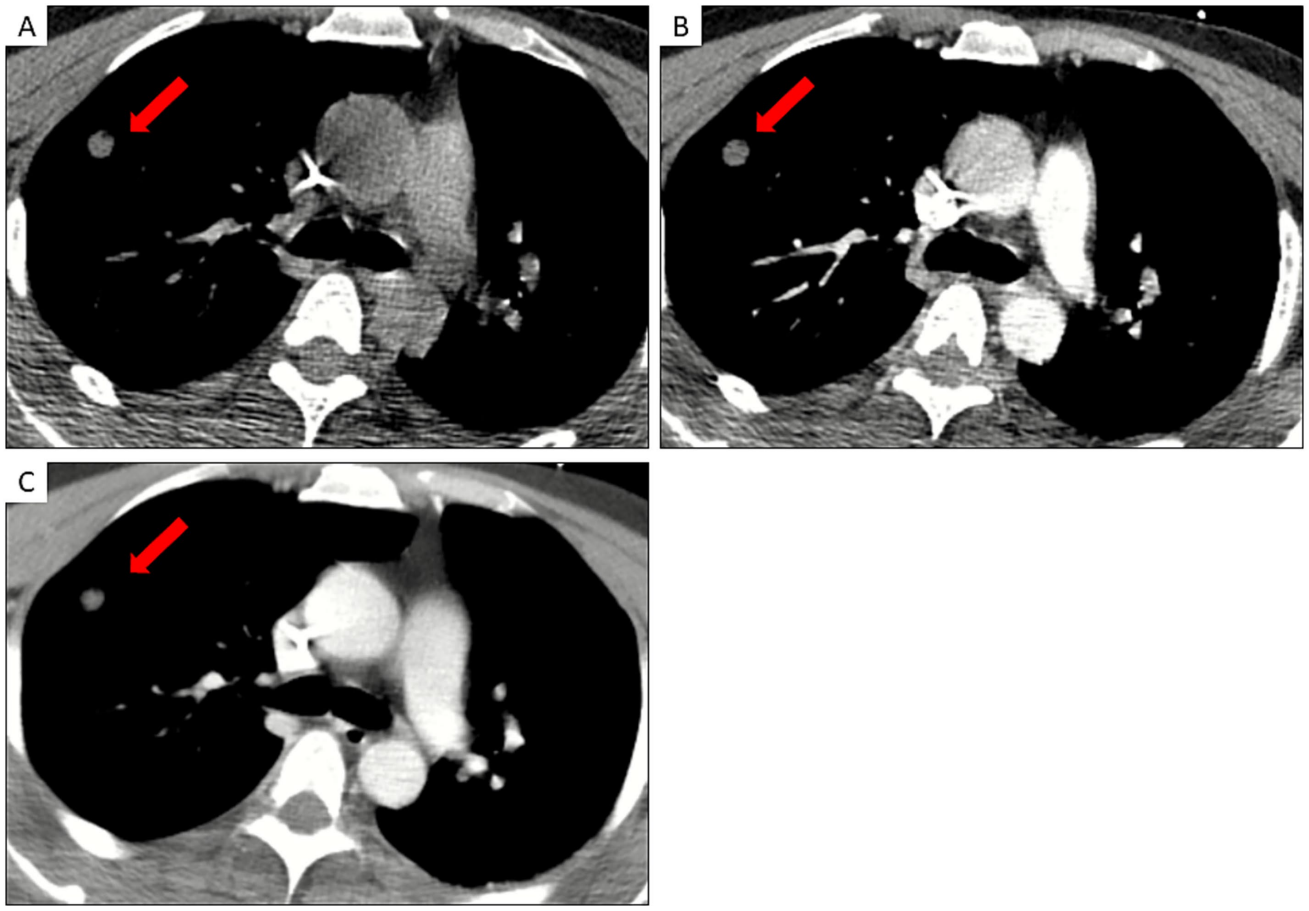
24-year-old male patient. **(A)** thin-layer CT mediastinal window of the anterior segmental nodular shadow in the upper lobe of the right lung; **(B,C)** CT contrast-enhanced arterial and delayed phases with no change in CT values of the anterior segmental nodule in the right upper lobe of the lung.

Nine patients with normal serum creatinine levels (range: 0.74–1.35 mg/dL) and no history of iodine or contrast agent allergy underwent dynamic contrast-enhanced CT after providing written informed consent. Across 76 nodules (CT attenuation: −200.4 to 310.0 HU; diameter: 2.1–27.1 mm), lesions were present in all lobes. CT attenuation values were measured in identical ROI regions before and after enhancement in the mediastinal window, and the enhancement difference was calculated. None of the nodules demonstrated enhancement (Table 4, Figure 3).

**Table 4:** The analysis of enhancement characteristics of 76 pulmonary nodules.

Characteristics	Nodule
Preenhancement attenuation (HU)	
Mean ± SD	53.07 ± 146.63
Median	58.05
Interquartile range (25–75%)	72.0–161.1
Range	−200.4 to 310.0
Enhancement (HU)	
Mean ± SD	5.75 ± 3.40
Median	5.05
Interquartile range (25–75%)	2.88–8.58
Range	0 to 11.9
Size (mm)	
Mean ± SD	14.34 ± 7.29
Median	14.95
Interquartile range (25–75%)	7.6–20.9
Range	2.1 to 27.1
No. of nodules in left lung	
Upper lobe	7 (9.2)
Lower lobe	19 (25)
No. of nodules in right lung	
Upper lobe	11 (14.5)
Middle lobe	21 (27.6)
Lower lobe	18 (23.7)

Numbers in parentheses are percentages.

**Figure 4:**
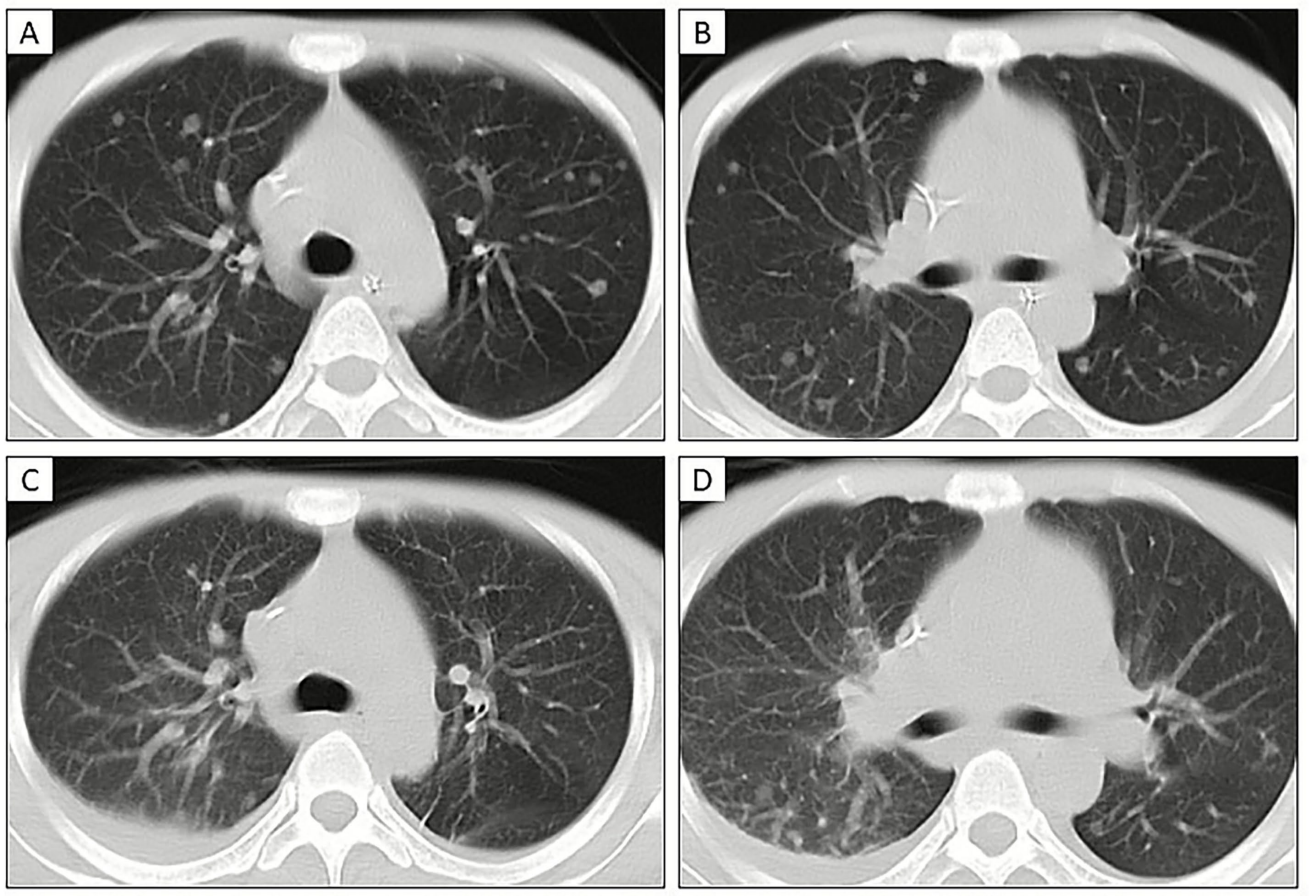
24-year-old man with pneumonia caused by Mpox who had genital skin rashes as initial symptoms. **(A,B)** CT axial scans show bilateral pulmonary solid nodular shadows; **(C,D)** following ART treatment, CT scans on October 9 show a reduction in the number of nodules, decreased density, and diminished ground-glass nodular shadows, with pleural effusion on the right side.

**Figure 5:**
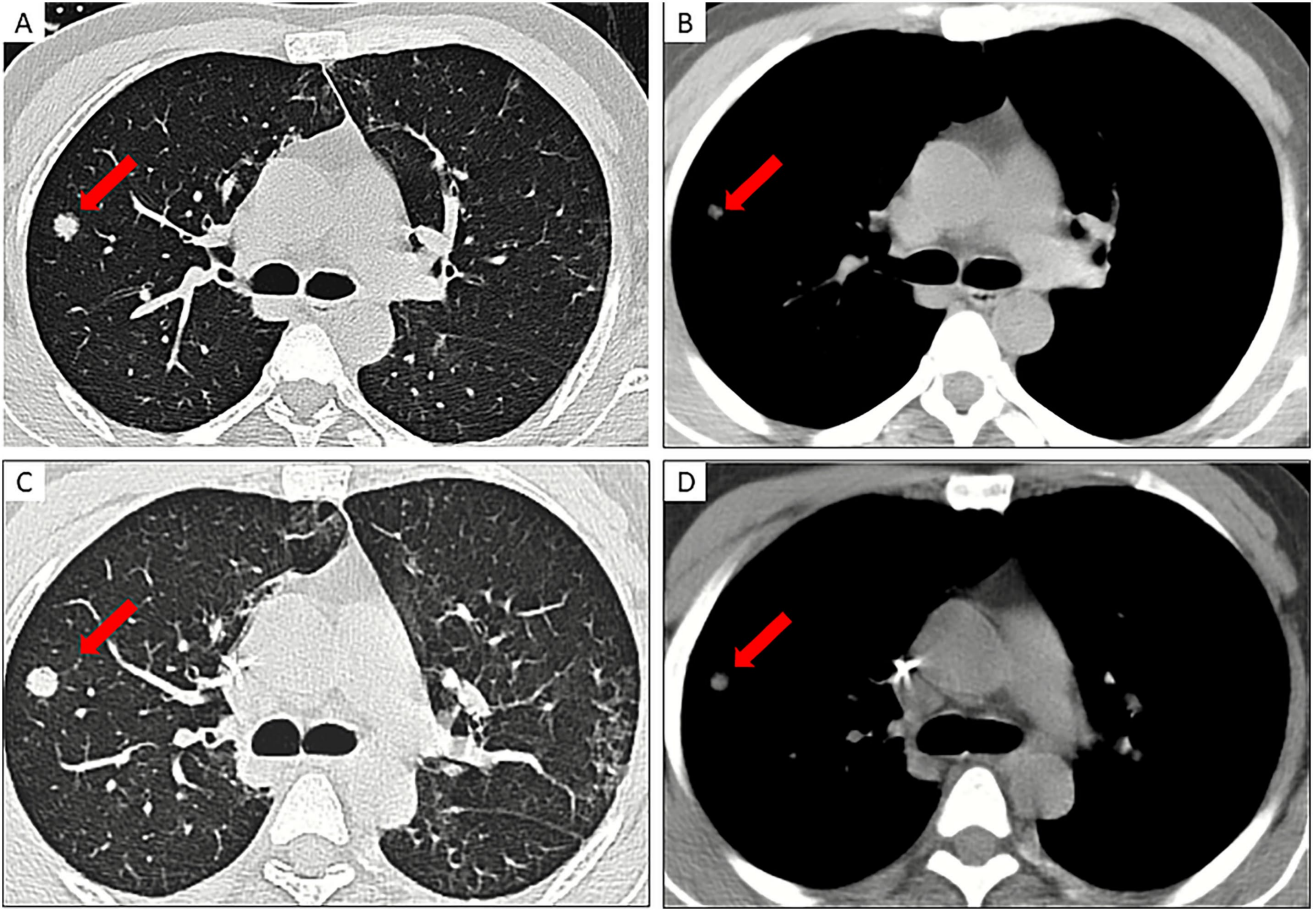
A 38-year-old male patient presented with Mpox pneumonia, initially manifesting symptoms of diarrhea and weakness. Twelve days prior to hospital admission, he engaged in unprotected homosexual intercourse. **(A,B)** chest CT scans with thin-section lung window (**A**, arrow) and mediastinal window (**B**, arrow) revealed a nodule in the anterior segment of the right upper lobe. **(C,D)** subsequent chest CT scans with thin-section lung window **(C)** and mediastinal window **(D)** indicated an increase in the size of the nodule, with greater solid component and heightened CT density of the right upper lobe nodule to 58 Hu, exhibiting more defined margins compared to the previous examination.

Among the 8 Mpox pneumonia patients who underwent longitudinal follow-up, three distinct radiological evolution patterns of pulmonary nodules were observed: (Kozlov, 2022) Resorptive pattern (*n* = 4): In four patients (follow-up durations: 31, 29, 20, and 19 days), CT during clinical recovery showed substantial reduction in the solid components of nodules, with complete resolution of some subcentimeter nodules, suggesting reversibility, as illustrated in Figure 4, World Health Organization (2023). Progressive pattern (*n* = 2): Two patients (follow-up at 40 and 16 days) exhibited increased nodule size and density during disease progression, indicating ongoing inflammation (Figure 5; Jadhav et al., 2025). Stable/calcified pattern (*n* = 2): In two patients (follow-up at 60 and 117 days), nodules remained radiologically stable with peripheral calcification, suggesting a transition to chronic repair or fibrosis. These findings suggest that the disease course of Mpox-related pulmonary solid nodules may be influenced by immune status, pathogen burden, and secondary infections. Clinically, enhanced dynamic imaging follow-up, combined with a comprehensive, systematic, intensified treatment approach, may help improve patient outcomes.

### Interobserver agreement

The interobserver agreement for radiologic features of nodules was as follows: Substantial agreement was observed in the identification of nodule lobulation (Cohen’s *κ* = 0.800, 95% CI: 0.586–1.014), spiculation (*κ* = 0.867, 95% CI: 0.693–1.045), mediastinal or hilar lymphadenopathy (*κ* = 0.809, 95% CI: 0.726–1.024), pleural effusion (*κ* = 0.800, 95% CI: 0.586–1.014), and pleural thickening (*κ* = 0.800, 95% CI: 0.586–1.014). Weighted kappa coefficients also demonstrated good agreement for nodule distribution by lung lobe (*κ* = 0.819, 95% CI: 0.671–0.968), number of involved lobes (*κ* = 0.819, 95% CI: 0.671–0.968), craniocaudal (vertical) distribution (*κ* = 0.819, 95% CI: 0.671–0.968), axial distribution (*κ* = 0.819, 95% CI: 0.671–0.968), and nodule composition (*κ* = 0.819, 95% CI: 0.671–0.968).

## Discussion

This multicenter study of HIV-infected patients in China found that Mpox pneumonia occurred almost exclusively in individuals with CD4^+^ T-cell counts <200/μL. Its hallmark CT features were multiple, well-demarcated, non-enhancing hematogenously disseminated solid nodules, often accompanied by abnormal inflammatory markers, multisystem involvement, and a higher risk of in-hospital mortality. These imaging features differ markedly in morphology, distribution, and vascular characteristics from those seen in common opportunistic infections such as pneumocystis jirovecii pneumonia, cytomegalovirus, and cryptococcosis. Such focal pulmonary findings may serve as sentinel signals of systemic dissemination, underscoring their critical role in the early identification of high-risk patients. Importantly, consistent and effective ART was strongly associated with improved outcomes, highlighting the pivotal role of early, sustained, and effective ART in reducing the risk of severe disease and mortality in Mpox pneumonia.

### Clinical characteristics and research focus

A total of 41 individuals with HIV infection were enrolled in this study, including 21 patients with Mpox-associated pneumonia and CD4^+^ T-cell counts <200/μL, and 20 HIV/Mpox co-infected individuals without pulmonary involvement who served as controls. All patients were male and reported a clear history of men who have sex with men (MSM) exposure. Most patients exhibited typical early cutaneous manifestations of Mpox infection, predominantly rash, often accompanied by fever, headache, sore throat, myalgia, fatigue, and lymphadenopathy—features consistent with previous reports (Suárez Rodríguez et al., 2022). The rash typically originated in the genital or perianal areas, suggesting transmission through close physical contact and reflecting the predominant sexual transmission route in the current outbreak (Thornhill et al., 2022).

In the control group of 20 HIV/Mpox co-infected patients without pulmonary involvement, most (18/20) followed a classical viral illness trajectory: systemic symptoms preceded or occurred concurrently with rash, representing prodromal or early-stage manifestations (Nalca et al., 2005; Giradkar and Khatib, 2022). In contrast, the 21 severely immunosuppressed individuals (CD4^+^ T-cell counts <200/μL) exhibited more complex and heterogeneous clinical trajectories: four developed fever 8–10 days after rash onset, and 13 remained afebrile within three weeks of rash appearance (five of whom developed fever during treatment after the third week of illness), suggesting delayed immune responses or attenuated inflammatory cascades. Autopsy studies have reported markedly reduced inflammatory cell infiltration and diminished local immune activation in Mpox lesions among AIDS patients (Ritter et al., 2024). Notably, although most controls had preserved immunity, one individual with a CD4^+^ T-cell counts <200/μL displayed a similar clinical pattern to the Mpox pneumonia group, remaining asymptomatic systemically for 3 weeks after rash onset, indicating Mpox progression consistent with immunosuppressed states. Moreover, immunosuppressed patients frequently developed severe multisystem complications, including necrotic genital and perianal lesions, necrotizing fasciitis and gangrene of the limbs, severe proctitis, bowel obstruction or perforation, sepsis, and septic shock. These findings reflect a systemic and fulminant disease course, with similar multi-organ involvement reported in prior literature (Mitjà et al., 2023; Thornhill et al., 2022; Basgoz et al., 2022; Cash-Goldwasser et al., 2022), warranting heightened clinical vigilance, particularly for early stratification and targeted intervention planning. Among these multisystem manifestations, pulmonary nodular lesions are particularly distinctive and readily identifiable by imaging, meriting focused investigation of their characteristics and underlying mechanisms.

### Pulmonary imaging features and mechanistic insights

Among the 21 HIV-infected individuals with CD4^+^ T-cell counts <200 cells/μL enrolled in this study, CT commonly revealed hematogenous disseminated solid nodules, which represent the principal radiological hallmark of Mpox pneumonia. These nodules were typically multiple, well-defined, and randomly distributed, with diameters mostly <30 mm. Contrast-enhanced CT revealed no enhancement in any lesion, indicating poor vascularization and supporting a necrotic nature. Autopsy findings further corroborate the pathological basis of these nodules: Mpox-associated pulmonary lesions are characterized by parenchymal necrosis, thrombosis, vasculitic injury, and pulmonary infarction, with relatively mild local inflammatory responses (Mitjà et al., 2023; Ritter et al., 2024; Duarte-Neto et al., 2023). Animal studies further confirm the hematogenous dissemination mechanism: following intravenous inoculation, Mpox virus spreads to pulmonary tissue via the bloodstream, accumulates in capillary endothelial cells and alveolar macrophages, and induces inflammatory infiltration, alveolar edema, and focal tissue necrosis (Johnson et al., 2011). Radiologically, some nodules exhibited lobulated contours or spiculated margins, potentially reflecting perilesional inflammation involving small vessels, lymphatics, or bronchioles (Jiang et al., 2023; Chu et al., 2016; Xiao et al., 2021). Furthermore, a study using [18F]-FDG PET/CT revealed that Mpox pulmonary nodules demonstrate mild metabolic activity, consistent with the low-grade inflammatory profile of necrotic lesions (Manta et al., 2023). Longitudinal imaging follow-up indicated that some nodules showed reduced solid components or complete resolution during recovery, suggesting partial reversibility; a few nodules exhibited increased density and calcification, likely reflecting lesion organization or chronic reparative processes.

Additionally, over half of the patients exhibited imaging features of concomitant infections alongside Mpox-specific nodules. These included bronchiolar inflammation, “tree-in-bud” patterns, patchy consolidation, and ground-glass opacities—indicative of potential coexisting opportunistic infections. The presence of pleural effusion further supports that these severely immunocompromised patients are susceptible to mixed pneumonia, exacerbating pulmonary dysfunction and systemic inflammatory responses. These findings underscore the need for heightened clinical awareness of possible pathogen co-infection in Mpox pneumonia, and the importance of early microbiological screening and comprehensive therapeutic interventions.

### Immunological status and prognostic implications

This study found that Mpox pneumonia predominantly affects HIV-infected individuals with severe immunosuppression, particularly those with CD4^+^ T-cell counts <200 cells/μL. CT imaging consistently demonstrated characteristic hematogenous disseminated solid nodules, suggesting that the virus may have breached local barriers and disseminated hematogenously to the lungs, reflecting the progression of systemic infection. Although these nodules exhibited relatively mild local inflammatory responses, they were frequently accompanied by multi-organ dysfunction. Patients were more prone to developing systemic inflammatory response syndrome (SIRS), thereby significantly increasing disease severity and mortality risk (Johnson et al., 2011).

In this study, all 21 patients with Mpox pneumonia exhibited systemic dissemination, defined as the involvement of two or more extrapulmonary organ systems. For these critically ill patients, a multidimensional and comprehensive intensive therapeutic strategy was initiated immediately upon hospital admission. However, long-standing immune impairment and the short treatment duration resulted in insufficient immune reconstitution, limiting therapeutic efficacy. Some cases further developed immune reconstitution inflammatory syndrome (IRIS), which further exacerbated disease progression and organ injury. A total of eight patients (38.1%) died. All fatalities occurred among individuals with profound immunosuppression resulting from irregular ART use or prolonged ART interruption, or among newly diagnosed HIV patients who failed to initiate ART in a timely manner (late presenters), all with CD4^+^ T-cell counts <200/μL. In contrast, all patients on regular ART, although experiencing incomplete immune reconstitution and multisystem involvement, survived under intensive therapy without life-threatening complications. By contrast, most patients in the control group (75.0%) had consistent and effective ART, presented with milder disease, and all were successfully discharged. These findings align closely with current guideline recommendations that ART should be continued or initiated as early as possible during acute infection. They underscore the critical role of early, sustained, and effective ART, combined with Mpox-specific therapy, in reducing the risks of severe disease and mortality in Mpox pneumonia (O'Shea et al., 2022).

Laboratory findings further supported the systemic nature of Mpox pneumonia. Compared with controls, abnormalities in inflammation, coagulation, and renal function were more frequent in the Mpox pneumonia group: elevated CRP in 66.7% and elevated PCT in 19.0% of patients, suggesting progression to severe infection or sepsis in some cases; anemia in 52.4%; and elevated creatinine in 28.6%, indicating that the inflammatory response may have involved hematopoietic and renal systems. Prolonged prothrombin time and elevated D-dimer were observed concurrently in most patients, reflecting a potential hypercoagulable state or risk of disseminated intravascular coagulation (DIC). These abnormalities not only highlight the systemic nature of the pneumonia but also provide reliable indicators for the early clinical identification of severe cases. In addition, the disease course in the Mpox pneumonia group was significantly longer than in controls, possibly reflecting delayed viral clearance and impaired immune recovery, particularly among immunocompromised individuals.

In summary, these specific pulmonary “focal manifestations” may in fact serve as “sentinel signals” of systemic dissemination. Clinicians should incorporate CD4^+^ T-cell counts, viral load, inflammatory markers, and ART adherence into a comprehensive assessment framework to enable early identification and precise intervention in high-risk patients.

### Radiological differential diagnosis and clinical implications

**Figure 6:**
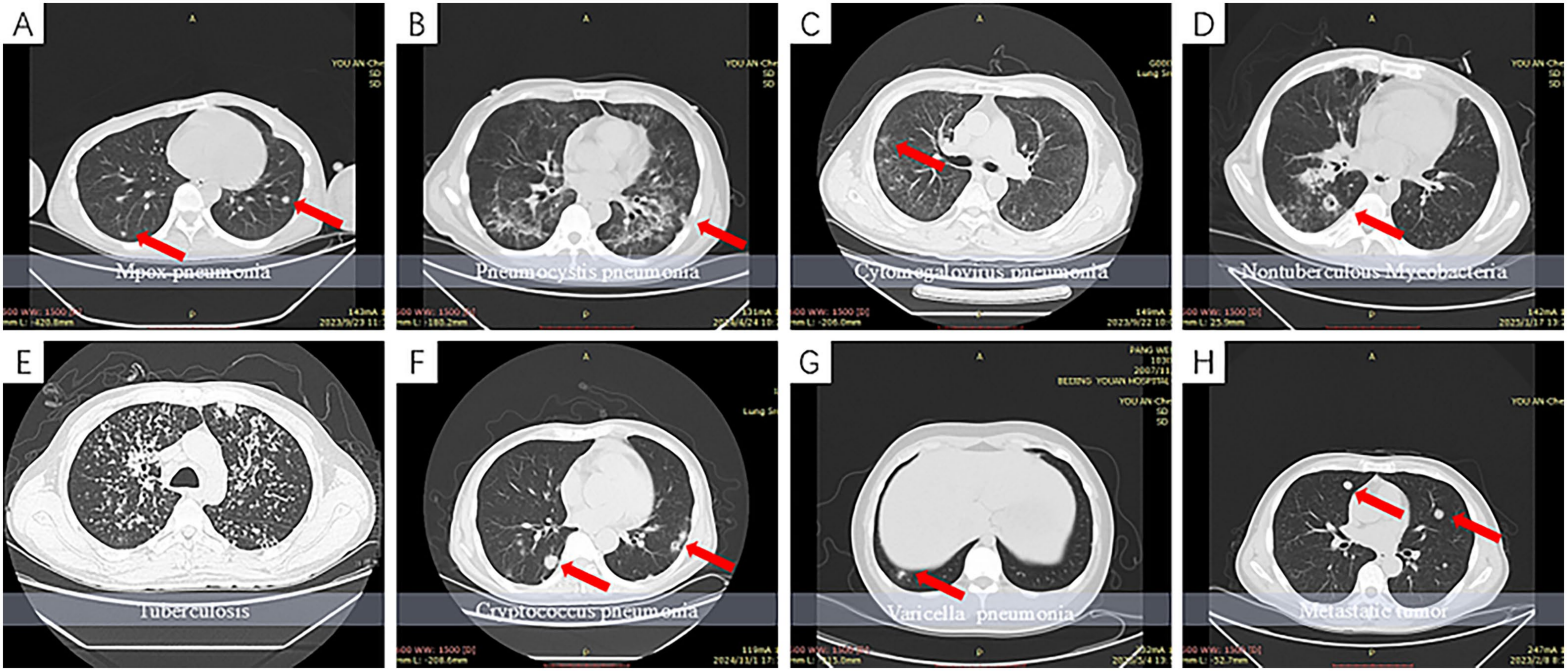
CT features of Mpox pneumonia and its major differential diagnoses in individuals with HIV infection. **(A)** Mpox pneumonia: multiple well-defined solid nodules with random distribution in both lungs, suggestive of hematogenous disseminated nodules. **(B)** Pneumocystis jirovecii pneumonia (PJP): symmetrically distributed diffuse ground-glass opacities predominantly around the hilar regions, reflecting exudative alveolitis. The nodules (indicated by the red arrow in the left lower lobe) show ill-defined margins and coexist with diffuse alveolar lesions. **(C)** Cytomegalovirus (CMV) pneumonia: patchy consolidations and ground-glass opacities distributed along the bronchovascular bundles, often accompanied by indistinct central micronodules (indicated by the red arrow in the right upper lobe), typically seen in a background of widespread inflammation. **(D)** Nontuberculous mycobacterial (NTM) infection: solid nodules in the right lower lobe showing cavitation (indicated by the red arrow) and a “tree-in-bud” appearance, commonly observed in patients with severe immunosuppression (CD4^+^ < 50 cells/μL). **(E)** Pulmonary tuberculosis: diffusely distributed bronchogenic satellite micronodules predominantly in the upper lung fields, with small cavitations within the lesions. **(F)** Cryptococcal pneumonia: multiple well-defined solid nodules distributed subpleurally in the lower lobes of both lungs (indicated by red arrows), some of which may show cavitation. **(G)** Varicella pneumonia: diffusely distributed sub-centimeter nodules throughout both lungs, often associated with the “halo sign.” **(H)** Pulmonary metastases: multiple solid nodules of varying sizes with well-defined margins, showing marked enhancement on contrast-enhanced CT. Although morphologically similar to Mpox nodules, these originate from hematogenous spread of solid tumors.

This study found that Mpox pneumonia predominantly occurs in HIV-infected individuals with impaired immune function, particularly those with markedly reduced CD4^+^ T-cell counts. Consequently, its radiological features should be interpreted within the context of HIV-related opportunistic infections and carefully differentiated from morphologically similar conditions, such as varicella-zoster pneumonia and pulmonary metastases, as illustrated in Figure 6.

#### Pneumocystis jirovecii pneumonia (PJP)

The typical CT findings of PJP include diffusely distributed ground-glass opacities with a symmetric pattern involving both lungs, reflecting exudative alveolitis. These lesions frequently affect the perihilar regions. In advanced stages, cystic changes, pneumothorax, or nodular lesions may occur; however, the nodules usually exhibit ill-defined margins and coexist with diffuse alveolar disease, forming a characteristic radiologic pattern (Kanne et al., 2012). This presentation is markedly different from the diffusely distributed, well-circumscribed solid nodules seen in Mpox pneumonia.

#### Cytomegalovirus (CMV) pneumonia

On CT, CMV pneumonia typically presents as patchy areas of consolidation accompanied by ground-glass opacities along the bronchovascular bundles. Small nodules, the “halo sign,” and subpleural irregularities are frequently observed, indicating involvement of both alveolar and interstitial compartments (Du et al., 2020; Moon et al., 2000). CMV-related nodules are often embedded within a background of extensive inflammation, exhibit irregular morphology, and are commonly associated with bronchial wall thickening and reticular interstitial changes (Moon et al., 2000). In contrast to the hematogenous, necrotic pulmonary nodules caused by Mpox, CMV nodules are more suggestive of a progressive and actively inflammatory process.

#### Tuberculosis and nontuberculous mycobacterial (NTM) infections

Both pathogens are common opportunistic infections among individuals with HIV, and their imaging features are markedly influenced by the host’s immune status. In immunocompetent patients, pulmonary tuberculosis typically presents as cavitary infiltrates predominantly in the upper lobes. In contrast, immunocompromised individuals—particularly those with markedly reduced CD4^+^ T cell counts—are more likely to exhibit consolidation in the lower lobes, symmetric parenchymal involvement, interstitial thickening, and mediastinal lymphadenopathy (Haramati et al., 1997). NTM infections are also frequently observed in patients with HIV/AIDS, especially when CD4^+^ T cell counts fall below 50 cells/μL (Lee et al., 2022). CT findings are heterogeneous but often include centrilobular nodules distributed along the bronchi and bronchovascular bundles, the “tree-in-bud” sign, with or without bronchiectasis, focal consolidation, atelectasis, and thin-walled cavities (Kim et al., 2017). NTM-associated nodules tend to be ill-defined, of solid or subsolid attenuation, and exhibit chronic progression with notable variability between species (Biciusca et al., 2024). These characteristics are in stark contrast to the well-defined, randomly distributed hematogenous necrotic nodules observed in Mpox pneumonia.

#### Cryptococcal pneumonia

As a common opportunistic infection among individuals with HIV, cryptococcal pneumonia on CT typically presents as solitary or multiple solid nodules with relatively well-defined margins, and occasionally cavitation. These nodules are predominantly located in the peripheral or subpleural regions of both lungs (Hu et al., 2017), in contrast to the hematogenous, randomly distributed necrotic solid nodules observed in Mpox pneumonia. In individuals with more profound immunosuppression, cryptococcal pneumonia may also manifest with atypical imaging features, such as diffuse interstitial infiltrates, consolidation, and pleural reactions. Comprehensive assessment should incorporate immune markers such as CD4^+^ T cell counts (Miller et al., 1990; Lacomis et al., 2001).

#### Varicella pneumonia

Caused by a virus from the same Poxviridae family as Mpox, varicella pneumonia typically appears on CT as diffusely distributed subcentimeter nodules in both lungs, often accompanied by a “halo sign” and characterized by a degree of self-limiting resolution. Imaging abnormalities generally resolve rapidly in parallel with the resolution of skin lesions (Abdelghani et al., 2009; Kim et al., 1999; Kim and Lee, 2020). In contrast, Mpox-associated pulmonary nodules are more frequently observed in patients with AIDS, exhibit well-defined borders, lack the “halo sign,” and, in some cases, show no reduction—or even increased density—during short-term follow-up. These findings suggest distinct underlying pathophysiological mechanisms from varicella, with less propensity for spontaneous resolution.

#### Pulmonary metastases

Radiologically, pulmonary metastases and Mpox-related pulmonary nodules can both present as multiple, well-circumscribed, round solid nodules distributed throughout both lungs with variable sizes and random distribution, often leading to diagnostic confusion. However, the two entities differ markedly in pathological basis and clinical context. Metastases typically result from hematogenous spread of a primary malignancy, and the nodules tend to be vascularized solid lesions that show significant enhancement on contrast-enhanced CT. In contrast, Mpox-related pulmonary nodules generally lack enhancement, reflecting their ischemic and necrotic nature, which may aid in preliminary differentiation during contrast imaging.

In summary, Mpox-related pulmonary nodules exhibit certain radiologic characteristics that may aid in their identification. A comprehensive differential diagnosis should incorporate features such as distribution pattern, nodule morphology, enhancement behavior, and clinical context, in order to avoid misdiagnosis or missed diagnosis and to inform appropriate treatment decisions and follow-up strategies.

This multicenter retrospective analysis offers preliminary insights into the imaging characteristics of Mpox pneumonia in people living with HIV (PLWH), yet several limitations remain. First, the relatively small sample size may limit the statistical power and generalizability of the findings. Second, although typical pulmonary nodules were predominantly observed in patients with CD4^+^ T cell counts <200 cells/μL, the specific impact of immune status on nodule characteristics and prognosis remains incompletely understood. Future studies should expand the sample size and adopt more refined immune and clinical stratification, incorporating comorbidities, the extent of pulmonary involvement, and the degree of multi-organ damage to comprehensively elucidate the relationships among immunosuppression, clinical background, imaging features, and clinical outcomes. Furthermore, PET/CT and histopathological evaluation may provide additional insights into the metabolic activity and evolution of these nodules. Prospective multicenter studies are warranted to establish an integrated imaging–immunity–pathology database, supported by artificial intelligence tools, to facilitate early identification and personalized management of patients with severe Mpox.

## Conclusion

This multicenter study is the first to systematically characterize the radiological features of Mpox pneumonia under immunosuppressive conditions. Patients with CD4^+^ T-cell counts <200/μL were most susceptible, typically presenting on CT with hematogenously disseminated necrotizing nodules accompanied by systemic inflammation and multisystem injury. These focal pulmonary manifestations may serve as early “sentinel signals” of systemic dissemination and hold potential value for radiological differentiation. Furthermore, consistent, sustained, and effective ART was associated with significantly improved outcomes, whereas patients with irregular or absent ART prior to admission experienced markedly higher mortality. These findings underscore the importance of early identification and immune status assessment in the context of concurrent Mpox and HIV epidemics, and highlight the necessity of implementing a comprehensive, systematic, intensified treatment approach to reduce the risk of severe disease and mortality.

## Data Availability

The original contributions presented in the study are included in the article/supplementary material, further inquiries can be directed to the corresponding authors.
